# Accuracy of Diagnostic Tests for the Detection of Chagas Disease: A Systematic Review and Meta-Analysis

**DOI:** 10.3390/diagnostics12112752

**Published:** 2022-11-10

**Authors:** Mayron Antonio Candia-Puma, Laura Yesenia Machaca-Luque, Brychs Milagros Roque-Pumahuanca, Alexsandro Sobreira Galdino, Rodolfo Cordeiro Giunchetti, Eduardo Antonio Ferraz Coelho, Miguel Angel Chávez-Fumagalli

**Affiliations:** 1 Computational Biology and Chemistry Research Group, Vicerrectorado de Investigación, Universidad Católica de Santa María, Arequipa 04000, Peru; 2 Facultad de Ciencias Farmacéuticas, Bioquímicas y Biotecnológicas, Universidad Católica de Santa María, Arequipa 04000, Peru; 3 Laboratório de Biotecnologia de Microrganismos, Universidade Federal São João Del-Rei, Divinópolis 35501-296, MG, Brazil; 4 Laboratório de Biologia das Interações Celulares, Instituto de Ciências Biológicas, Universidade Federal de Minas Gerais, Belo Horizonte 31270-901, MG, Brazil; 5 Instituto Nacional de Ciência e Tecnologia em Doenças Tropicais, INCT-DT, Salvador 40015-970, BA, Brazil; 6 Programa de Pós-Graduação em Ciências da Saúde, Infectologia e Medicina Tropical, Faculdade de Medicina, Universidade Federal de Minas Gerais, Belo Horizonte 31270-901, MG, Brazil; 7 Departamento de Patologia Clínica, COLTEC, Universidade Federal de Minas Gerais, Belo Horizonte 31270-901, MG, Brazil

**Keywords:** Chagas disease, diagnostic tests, meta-analysis, systematic review, sensitivity and specificity

## Abstract

The present systematic review and meta-analysis about the accuracy of diagnostic tests aim to describe the findings of literature over the last thirty years for the diagnosis of Chagas disease (CD). This work aimed to determine the accuracy of diagnostic techniques for CD in the disease’s acute and chronic phases. The PubMed database was searched for studies published between 1990 and 2021 on CD diagnostics. Fifty-six published studies that met the criteria were analyzed and included in the meta-analysis, evaluating diagnostic accuracy through sensitivity and specificity. For Enzyme-Linked Immunosorbent Assay (ELISA), Fluorescent Antibody Technique (IFAT), Hemagglutination Test (HmT), Polymerase Chain Reaction (PCR), and Real-Time Polymerase Chain Reaction (qPCR) diagnosis methods, the sensitivity had a median of 99.0%, 78.0%, 75.0%, 76.0%, and 94.0%, respectively; while specificity presented a median of 99.0%, 99.0%, 99.0%, 98.0%, and 98.0%, respectively. This meta-analysis showed that ELISA and qPCR techniques had a higher performance compared to other methods of diagnosing CD in the chronic and acute phases, respectively. It was concluded utilizing the Area Under the Curve restricted to the false positive rates (AUC_FPR_), that the ELISA diagnostic test presents the highest performance in diagnosing acute and chronic CD, compared to serological and molecular tests. Future studies focusing on new CD diagnostics approaches should be targeted.

## 1. Introduction

Chagas disease (CD) is an *anthropozoonosis* caused by the protozoan parasite *Trypanosoma cruzi*, which is transmitted mainly by blood-sucking bugs (also known as the *“kissing-bug”*) from the subfamily *Triatominae* [1,2] Other transmission forms are vertical transmission from mother to child or by blood transfusion, organ transplant, laboratory accident, oral contamination, and breastfeeding [3]. Over six million people are affected by the disease in Latin America, being endemic in 21 countries [4,5,6]. Additionally, it has been proposed that in the United States approximately 300,000 persons live with the infection, including 57,000 Chagas cardiomyopathy patients and 43,000 infected reproductive-age women [7], even though only a small fraction are properly diagnosed and treated [8]. Comparably, in the last decade, globalization has allowed the disease to spread through European countries, such as Austria, Belgium, France, Germany, Italy, Netherlands, Portugal, Spain, Sweden, Switzerland, the United Kingdom, Australia, Japan, and Canada [9,10]. In this scenario, at least 100 million people have a high risk of infection by living in endemic areas of disease [11], while the estimated annual global burden is $627.46 million in healthcare costs and 800,000 disability-adjusted life-years [7,12], besides that, approximately 10,000 deaths per year can be attributed to the disease [13], making CD a serious public health problem worldwide.

The infection has two distinct clinical phases separated by an indeterminate period [14]. In the acute phase, the disease is characterized by high parasitemia; usually asymptomatic or oligosymptomatic; and patients can exhibit fever, anorexia, tachycardia, and cutaneous manifestations, such as *Chagoma’s* and *Romaña’s* signs [15]; In the chronic phase, the infection can manifest as neurological, cardiac, digestive, and cardio-digestive alterations [16]. CD cardiomyopathy is the most severe and life-threatening manifestation of the disease; affecting about 40% of patients in the chronic phase and appearing as heart failure, arrhythmia, heart block, thromboembolism, stroke, and sudden death [12]. Mega-visceral syndromes are caused by denervation of the enteric nervous system that appears years after the acute infection and includes Megacolon (the commonest form), Megaesophagus, and Chagasic enteropathy, where about 10% of asymptomatic patients with chronic CD have radiological gastrointestinal abnormalities [13], as well, the neurological manifestations of CD manifests as neuritis, which results in distorted tendon reflexes and sensory impairment, while is reported in up to 10% of the patients. Isolated central nervous system involvement cases can also include dementia, confusion, chronic encephalopathy, and sensitive and motor deficits [17].

Control strategies applied to CD combine two approaches, which include the prevention, diagnosis, and treatment of infected individuals [18]; however, despite being a disease that has been discovered more than a century ago, there is little access to diagnosis and treatment in primary health care [19,20]. Benznidazole (BNZ) and Nifurtimox (NFX) are employed currently as drugs in the therapy against CD, while BNZ treatment is counter-indicated for pregnant women and people with significant hepatic and renal illness; NFX is recommended as a second-line drug, only in cases where BNZ failures and in absence of neurological and psychiatric disorders [21]; likewise, both are not effective in the chronic phase of the disease and produce severe adverse reactions [22]. Otherwise, potential vaccine candidates against CD have been investigated in recent decades; however, none have passed the preclinical stages of development. [23,24].

Laboratory diagnostic tests for CD depend largely on the clinical stage of the disease, while in the acute phase, it allows the direct detection of the parasite using molecular biology (polymerase chain reaction and real-time polymerase chain reaction) or parasitological (xenodiagnoses) techniques; oppositely, in the chronic phase, parasitemia becomes low and intermittent [25,26,27,28]; still, acute infection leads to seroconversion and anti-*T. cruzi*-specific immunoglobulins are detectable for years, so the infection can be indirectly identified by serological methods, such as enzyme-linked immunosorbent assay (ELISA), complement fixation test (CFT), fluorescent antibody technique (IFAT), hemagglutination test (HmT), radioimmunoprecipitation assay (RIPA), and western blot (WB) [29]. However, at present, there is no gold standard diagnostic test, since commercial tests have shown a high rate of false-positive results [30], for this reason, the World Health Organization (WHO) recommends that the diagnosis of CD should be carried out using two conventional tests based on the detection of different antigens [31]; and in the case of ambiguous or discordant results, a third technique should be used [32]. This situation reveals the urgent need for the development of new diagnostic tools for disease diagnosis [33,34,35]. A satisfactory method will allow the establishment of a patient registry with CD, a useful tool to provide information on its epidemiology, characteristics, and treatment [36]. Additionally, it must be considered that a behavioral design that allows establishing the reasons for people’s refusal to participate in diagnostic campaigns for this disease can alter the internal and external validity of the diagnosis [33]. 

The objective of the current work is to systematically review and summarize the available literature on the diagnostic accuracy of diagnostic tests for CD. For this, a systematic review of the medical literature was carried out over the period from 1990 to 2021. Results were analyzed through a meta-analysis based on the techniques used in diagnosing CD. The diagnostic techniques examined were PCR, qPCR, Xenodiagnosis, ELISA, CFT, IFAT, HmT, RIPA, and WB. Thus, we hope that the data generated will help to identify the basic need to fund the research organization for the screening, improvement, and effectiveness of CD diagnosis.

## 2. Materials and Methods

### 2.1. Search Strategy

This research was developed following the Preferred Reporting Items for Systematic Reviews and Meta-Analyses (PRISMA) guidelines [34], and the review was registered in the International Platform of Registered Systematic Review and Meta-analysis Protocols (INPLASY). The registration number is INPLASY202290132. This study met all recommended items reported by the PRISMA 2020 checklist (Table S1) [34].

The systematic review search strategy employed to evaluate the literature was developed as described [35]. PubMed provides uniform indexing of biomedical literature, the Medical Subject Headings (MeSH terms), which form a controlled vocabulary or specific set of terms that describe the topic of a paper consistently and uniformly [37], while selected terms were employed in a search carried out at the PubMed database (https://pubmed.ncbi.nlm.nih.gov/, last accessed on 5 July 2021). Although author keywords are particularly useful, the choice of terms can vary from paper to paper and from author to author. For this, MeSH terms were employed in the string query to improve the accuracy of the search. Pubmed is the main database for the health sciences, generated by the National Center for Biotechnology Information (NCBI) at the National Library of Medicine (NLM), the database contains about 32 million citations, belonging to more than 5300 journals currently indexed in MEDLINE [38]. While firstly, to find terms associated in the literature with CD diagnosis, the MeSH term *“Chagas Disease”* was employed alone, as the results were plotted into a network map of the co-occurrence of MeSH terms in the VOSviewer software (version 1.6.17) [39], which employs a modularity-based method algorithm to measure the strength of clusters [40]. The resulting cluster content was analyzed to select relevant terms associated with diagnostic techniques. Lastly, a second search was designed with each MeSH term obtained in the cluster analysis, associated with the MeSH term *“Sensitivity and Specificity”*, which are commonly regarded as summary points of diagnostic accuracy of a test [41], and the MeSH term *“Chagas Disease”*.

### 2.2. Study Selection and Data Extraction

The studies were selected in three stages. In the first, non-English language articles, duplicate articles, reviews, and meta-analyses were excluded; only articles published after 1990 and conducted on humans were included. In the second stage, the titles and abstracts of the articles selected through the search strategy were examined. Finally, the highly relevant full studies were retrieved and separated from the articles with a title or abstract that did not provide sufficient data to be included. The information consigned for each study chosen included the diagnostic technique, the number, type, and clinical characteristics of patients with CD and healthy controls. All studies evaluating the sensitivity and specificity of CD diagnostic techniques have been included. Furthermore, the data related to the geographical distribution, the number of studies carried out by country, and the frequency of the diagnostic techniques used were extracted. Studies with unclear or missing data regarding the CD and healthy control groups or data describing the sensitivity and specificity obtained in the studies were excluded from further analysis.

### 2.3. Statistical Analysis

Results were entered into Microsoft Excel (version 10.0, Microsoft Corporation, Redmond, WA, USA) spreadsheets and analyzed in the R programming environment (version 4.0.3) using the package *“mada”* (version 0.5.10) (https://cran.r-project.org/web/packages/mada/index.html (accessed on 8 April 2022); which employs a hierarchical model that accounts for within and between-study (heterogeneity) and the correlation between sensitivity and specificity [42]. Initially, the number of true negatives (TP), false negatives (FN), true positives (TP), and false positives (FP) were analyzed separately for each diagnostic technique; while the evaluation of sensitivity (Se) and specificity (Sp) made it possible to determine the diagnostic performance. Additionally, the positive likelihood ratio (LR+) expresses the ratio between the probability of expecting a positive test in a patient and the probability of expecting a positive test in a patient without the disease [43]; the negative likelihood ratio (LR−), which expresses the probability that a patient will test negative among people with the disease and the probability that a patient will test negative among people without disease; and the diagnostic likelihood ratio (DOR), which is the odds ratio of the positivity of a diagnostic test result in the diseased population relative to the non-diseased population [44]; and the 95% confidence interval (CI) were determined. Summary receiver operating characteristic (sROC) curves were fitted, according to the parameters of the *“Reitsma”* model of the *“mada”* package, and were used to compare the diagnostic accuracy of CD diagnostic techniques [45]. The confidence level for all calculations was set to 95%, using a continuity correction of 0.5 if pertinent.

## 3. Results

### 3.1. Search Results and Characteristics of the Selected Studies

**Figure 1:**
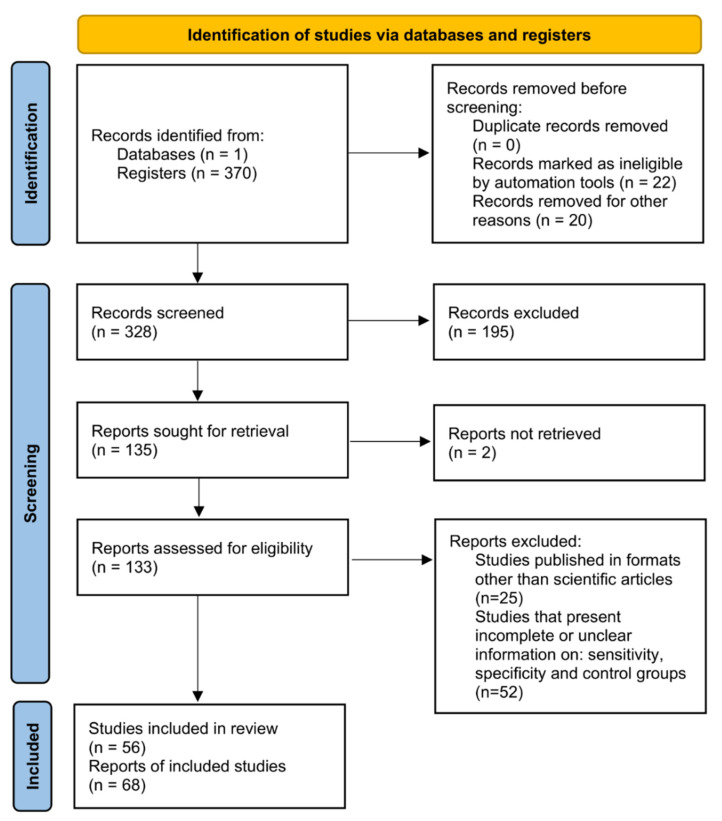
Systematic review and meta-analysis workflow diagram.

**Figure 2:**
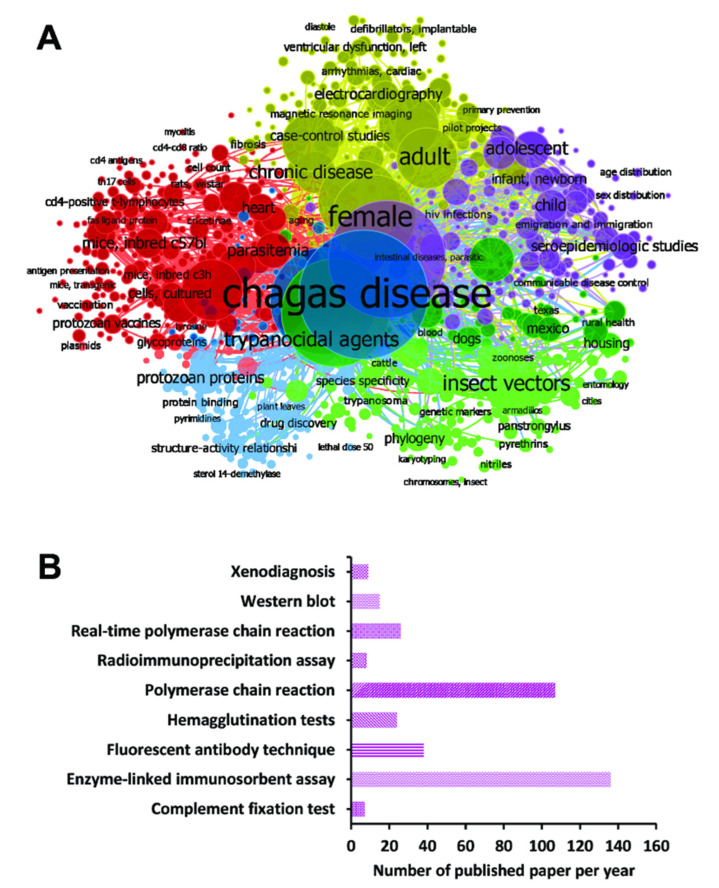
Papers were selected for the different diagnostic techniques using MeSH terms in the PubMed database. (**A**) Bibliometric map created by VOSviewer based on MeSH terms co-occurrence. (**B**) Number of papers found in the search for each diagnostic technique found in cluster analysis.

In the current work, a systematic review followed by a meta-analysis to measure the accuracy of diagnostic tests for CD was performed, and a flowchart of the strategy employed to select the studies is shown (Figure 1). For this, a search with the MeSH Terms *“Chagas Disease”* was performed in the Pubmed database, followed by the construction of a network map of the co-occurrence of MeSH terms; the search resulted in 370 published papers in a 1990–2021-year range, whereas establishing the value of five as the minimum number of occurrences of keywords, a map with 969 keywords that reaches the threshold was constructed (Figure 2A). In the analysis of the map, it is shown that five main clusters were formed, while terms such as “*Enzyme-linked immunosorbent assay*”, “*Polymerase chain reaction*”, “*Real-Time Polymerase Chain Reaction*”, “*Xenodiagnosis*”, “*Complement Fixation Tests*”, “*Fluorescent Antibody Technique*”, “*Hemagglutination Tests*”, “*Radioimmunoprecipitation Assay*”, and “*Blotting, Western*”, associated with diagnostic techniques were observed in the fifth cluster (purple color). Additionally, terms such as “*Chagas disease*”, “*humans*”, “*Trypanosoma cruzi*”, “*female*”, “*trypanocidal agents*”, “*adult*”, and “*insect vectors*” were recurrent denominators (Figure 2A).

The terms identified in this first analysis were employed in a second search at the Pubmed database, while associated each new term with the terms “*Chagas Disease*” and “*Sensitivity and Specificity*”; forming the new search strings: “*Chagas Disease*” [MeSH Terms] AND “*Sensitivity and Specificity*” [MeSH Terms] AND “*Polymerase Chain Reaction*” [MeSH Terms] for PCR; “*Chagas Disease*” [MeSH Terms] AND “*Sensitivity and Specificity*” [MeSH Terms] AND “*Real-Time Polymerase Chain Reaction*” [MeSH Terms] for qPCR; “*Chagas Disease*” [MeSH Terms] AND “*Sensitivity and Specificity*” [MeSH Terms] AND “*Xenodiagnosis*” [MeSH Terms] for XD; “*Chagas Disease*” [MeSH Terms] AND “*Sensitivity and Specificity*” [MeSH Terms] AND “*Enzyme-Linked Immunosorbent Assay*” [MeSH Terms] for ELISA; “*Chagas Disease*” [MeSH Terms] AND “*Sensitivity and Specificity*” [MeSH Terms] AND “*Complement Fixation Tests*” [MeSH Terms] for CFT; “*Chagas Disease*” [MeSH Terms] AND “*Sensitivity and Specificity*” [MeSH Terms] AND “*Fluorescent Antibody Technique*” [MeSH Terms] for IFAT; “*Chagas Disease*” [MeSH Terms] AND “*Sensitivity and Specificity*” [MeSH Terms] AND “*Hemagglutination Tests*” [MeSH Terms] for HmT; “*Chagas Disease*” [MeSH Terms] AND “*Sensitivity and Specificity*” [MeSH Terms] AND “*Radioimmunoprecipitation Assay*” [MeSH Terms] for RIPA; and “*Chagas Disease*” [MeSH Terms] AND “*Sensitivity and Specificity*” [MeSH Terms] AND “*Blotting, Western*” [MeSH Terms] for WB.

**Figure 3:**
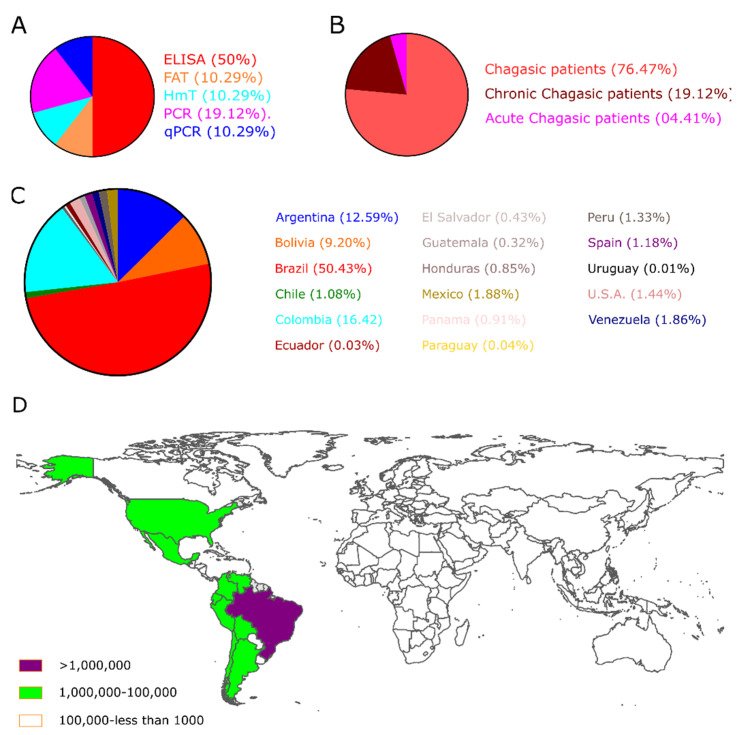
The geographical location of Chagas’s disease studies. (**A**) The pie chart shows the biomarkers; (**B**) the clinical description of patients, and (**C**) the Number of CD patients included in the selected studies worldwide. (**D**) Estimative of the global prevalence of Chagas disease, 2017 [8].

Besides, the number of selected studies for PCR, qPCR, Xenodiagnosis, ELISA, CFT, IFAT, HmT, RIPA, and WB was: 107, 26, 9, 136, 7, 38, 24, 8, and 15, respectively (Figure 2B). Though using a three-step eligibility criterion, 237 articles were excluded in the first two steps. In the last stage of data extraction, 41 articles were excluded. Therefore, 92 articles were selected for meta-analysis. Additionally, data on geographical characteristics extracted from the 92 selected articles were analyzed (Figure 3); while in most of the studies, the diagnostic technique employed was ELISA (Figure 3A); additionally, the American continent has carried out a greater number of studies, with Brazil being the country where a higher number of the population of Chagasic patients, in general, have been studied (Figure 3B–D).

### 3.2. Meta-Analysis of the Diagnostic Techniques for CD

#### 3.2.1. Enzyme-Linked Immunosorbent Assay

**Figure 4:**
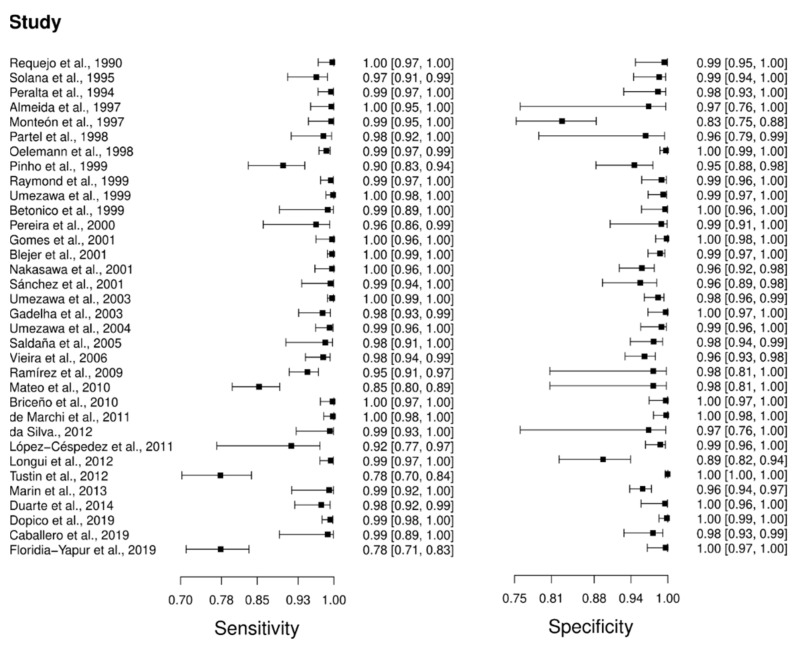
Study data and paired forest plot of the sensitivity and specificity of Enzyme-linked immunosorbent assay (ELISA) in Chagas’s disease diagnosis. Data from each study are summarized. Sensitivity and specificity are reported with a mean (95% confidence limits). The Forest plot depicts the estimated sensitivity and specificity (black squares) and its 95% confidence limits (horizontal black line) [46,47,48,49,50,51,52,53,54,55,56,57,58,59,60,61,62,63,64,65,66,67,68,69,70,71,72,73,74,75,76,77,78,79].

Thirty-four studies were selected for the ELISA technique: [46,47,48,49,50,51,52,53,54,55,56,57,58,59,60,61,62,63,64,65,66,67,68,69,70,71,72,73,74,75,76,77,78,79] in which a total of 6054 subjects were studied. Sensitivity ranged from 78.0% to 100%, with a median of 99.0%, CI 95% (94, 100), while the test for equality of sensitivities presented a χ^2^ = 657.24, df = 33, *p*-value = 2 × 10^−16^. Study specificity ranged from 83.0 to 100%, with a median of 99.0%, 95%CI (95, 100); the test for equality of specificities showed χ^2^ = 311.4699, df = 33, *p*-value = 2 × 10^−16^. Additionally, the results regarding LR+ {median 71.74, 95%CI (13.71, 476.73)}, LR− {median 0.01, 95%CI (0.00, 0.08)} and DOR {median 5938.93, 95%CI (331.76, 100,154.55)}. The analyzed diagnostic performance is summarized in Figure 4 and Figure S1.

#### 3.2.2. Fluorescent Antibody

**Figure 5:**
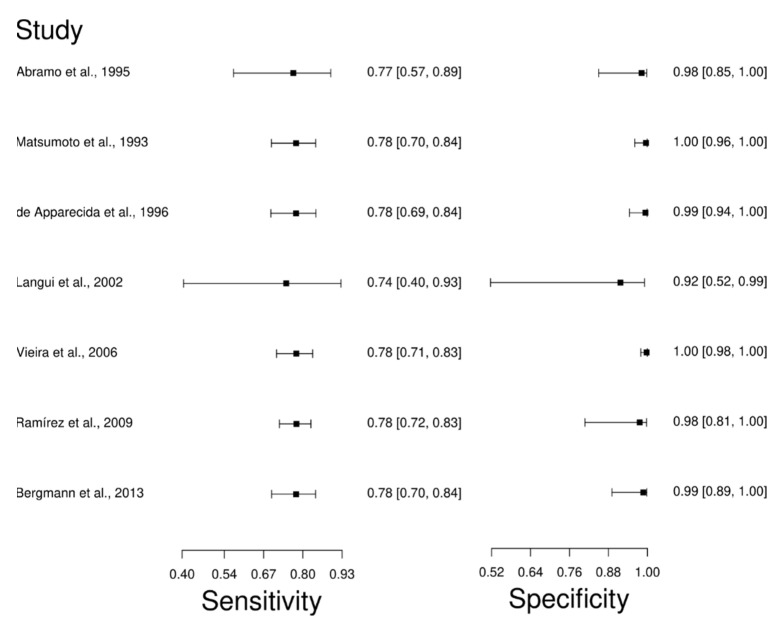
Study data and paired forest plot of the sensitivity and specificity of Fluorescence antibody assay (IFAT) in Chagas’s disease diagnosis. Data from each study are summarized. Sensitivity and specificity are reported with a mean (95% confidence limits). The Forest plot depicts the estimated sensitivity and specificity (black squares) and its 95% confidence limits (horizontal black line) [66,67,80,81,82,83,84].

Seven studies were selected for the IFAT diagnostic technique: [66,67,80,81,82,83,84]. A total of 810 subjects were studied. Sensitivity ranged from 74.0 to 78.0%, with a median of 78.0%, 95%CI (70, 84); while the test for equality of sensitivities showed: χ^2^ = 0.064, df =6, *p*-value = 1. The specificity of the studies ranged from 92.0 to 100%, with a median of 99.0%, 95%CI (89, 100); while the test for equality of specificities presented χ^2^ = 7.89, df = 6, *p*-value = 0.246. In addition, the results regarding LR+ {median 62.22, 95%CI (3.95, 979.16)}, LR− {median 0.23, 95%CI (0.16, 0.31)}, and DOR {median 62.22, 95%CI (3.95, 979.16)} are displayed. The analyzed diagnostic performances are summarized in Figure 5 and Figure S2.

#### 3.2.3. Hemagglutination Tests

**Figure 6:**
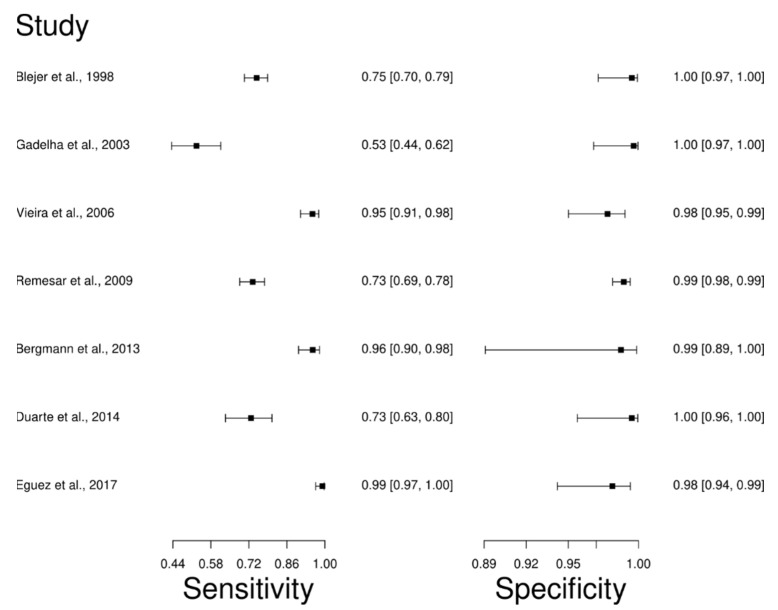
Study data and paired forest plot of the sensitivity and specificity Hemagglutination test (HmT) in Chagas’s disease diagnosis. Data from each study are summarized. Sensitivity and specificity are reported with a mean (95% confidence limits). The Forest plot depicts the estimated sensitivity and specificity (black squares) and its 95% confidence limits (horizontal black line) [63,66,76,84,85,86,87].

The analysis identified 24 published studies that used HmT as a diagnostic technique for CD. After analysis, only seven studies [63,66,76,84,85,86,87] were selected. A total of 1450 subjects were studied. The sensitivity of the studies ranged from 53.0 to 99.0%, with a median of 75.0%, and 95%CI (70, 80). Test for equality of sensitivities analysis showed: χ^2^ = 162.98, df = 6, *p*-value = 2 × 10^−16^. The specificity of the studies ranged from 98.0 to 100%, with a median of 99.0%, 95%CI (96, 100); while the test for equality of specificities: χ^2^ = 5.19, df = 6, *p*-value = 0.51. In addition, the results regarding LR+ {median 76.42, 95%CI (15.72, 1201.06)}, LR− {median 0.25, 95%CI (0.20, 0.30)} and DOR {median 633.85, 95%CI (76.19, 5332.02)} are displayed. The diagnostic performance of the selected studies is summarized in Figure 6 and Figure S3.

#### 3.2.4. Polymerase Chain Reaction

**Figure 7:**
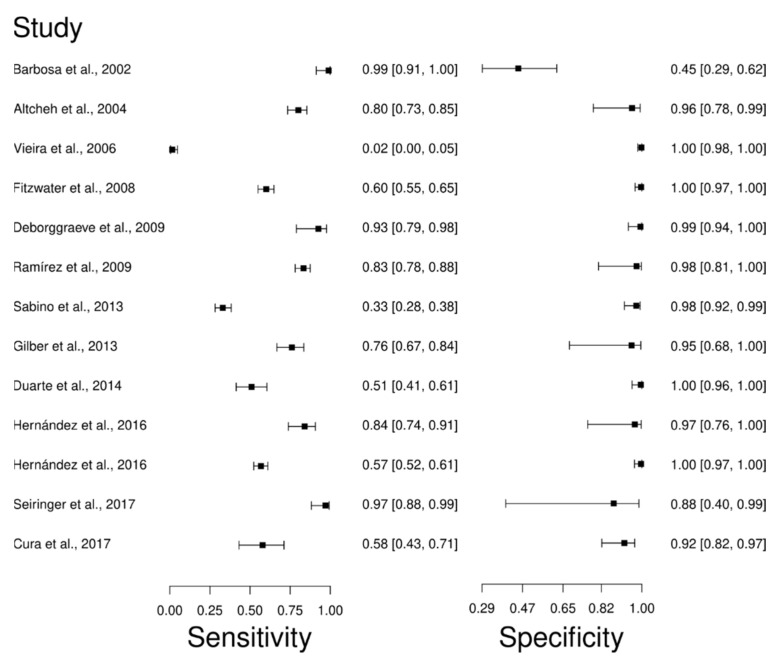
Study data and paired forest plot of the sensitivity and specificity Polymerase chain reaction (PCR) in Chagas’s disease diagnosis. Data from each study are summarized. Sensitivity and specificity are reported with a mean (95% confidence limits). The Forest plot depicts the estimated sensitivity and specificity (black circles) and its 95% confidence limits (horizontal black line) [66,67,76,88,89,90,91,92,93,94,95,96,97].

Thirteen studies were selected for the PCR diagnostic technique [66,67,76,88,89,90,91,92,93,94,95,96,97]. A total of 2198 subjects were studied. Sensitivity ranged from 2.0 to 99%, with a median of 76.0%, and CI of 95% (67, 84), while the test for equality of sensitivities presented a χ^2^ = 516.43, df = 12, *p*-value = 2 × 10^−16^. Study specificity ranged from 45.0 to 100%, with a median of 98.0%, 95%CI (82, 100); the test for equality of specificities showed χ^2^ = 315.74, df = 12, *p*-value = 2 × 10^−16^. In addition, the results regarding LR+ {median 18.32, 95% CI (2.59, 251.81)}, LR− {median 0.25, 95%CI (0.17, 0.36)}, and DOR {median 163, 95%CI (9.1, 2920.82)}. The analyzed diagnostic performance is summarized in Figure 7 and Figure S4.

#### 3.2.5. Real-Time Polymerase Chain Reaction

**Figure 8:**
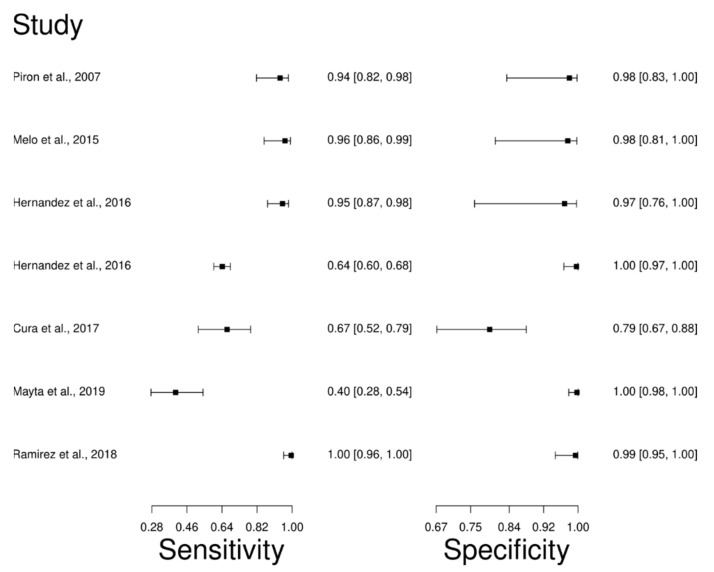
Study data and paired forest plot of the sensitivity and specificity quantitative Polymerase chain reaction (qPCR) in Chagas’s disease diagnosis. Data from each study are summarized. Sensitivity and specificity are reported with a mean (95% confidence limits). The Forest plot depicts the estimated sensitivity and specificity (black squares) and its 95% confidence limits (horizontal black line) [27,94,95,97,98,99,100].

The analysis identified 26 published studies that used qPCR as a diagnostic technique for CD. After analysis, only seven studies [27,94,95,97,98,99,100] were selected. A total of 995 subjects were studied. Sensitivity ranged from 40.0 to 100%, with a median of 94.0%, 95% CI (82, 98); while the test for equality of sensitivities showed: χ^2^ = 122.39, df =6, *p*-value = 2 × 10^−16^. The specificity of the studies ranged from 79.0 to 100%, with a median of 98.0%, 95% CI (83, 100); while the test for equality of specificities presented χ^2^ = 81.46, df = 6, *p*-value = 1.78 × 10^−15^. In addition, the results regarding LR+ {median 46.92, 95%CI (3.01, 730.43)}, LR− {median 0.06, 95%CI (0.02, 0.21)}, and DOR {median 597.2, 95%CI (31.35, 12131.7)} are displayed. The analyzed diagnostic performances are summarized in Figure 8 and Figure S5.

#### 3.2.6. Other Techniques

Regarding the Xenodiagnosis, CFT, RIPA, and WB diagnostic techniques, one [101], one [102], three [103,104], and three [105,106,107] studies were selected, respectively. While according to the criteria established in the workflow no analysis was performed regarding these diagnostic techniques.

#### 3.2.7. Summary ROC Curves (sROC)

**Figure 9:**
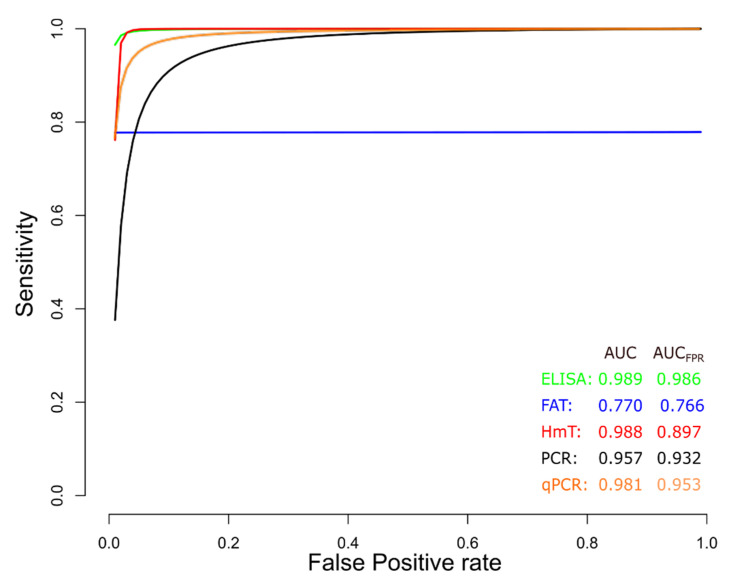
Meta-analysis of diagnostic test accuracy analysis. Summary receiver operating curve (sROC) plot of false positive rate and sensitivity. Comparison between ELISA, IFAT, HmT, PCR, and qPCR methods in the diagnosis of chronic Chagas disease.

**Figure 10:**
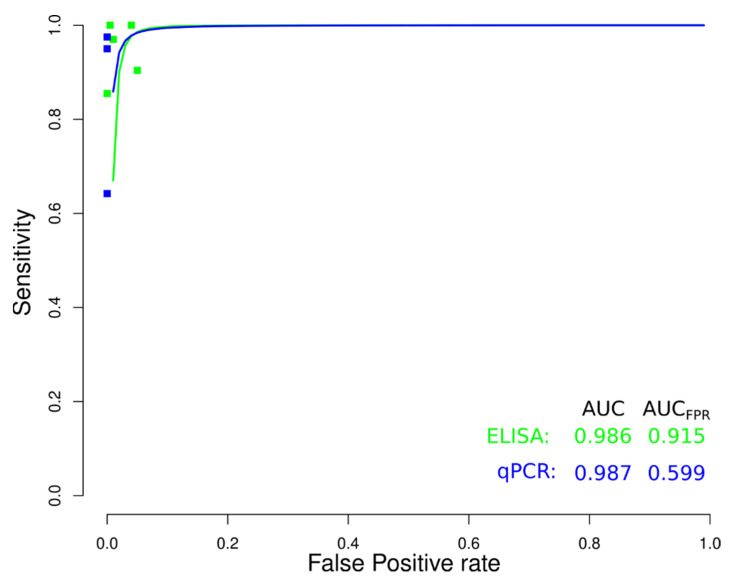
Meta-analysis of diagnostic test accuracy analysis. Summary receiver operating curve (sROC) plot of false positive rate and sensitivity. Comparison between ELISA and qPCR methods in the diagnosis of acute Chagas disease.

sROC curve analysis was conducted to compare diagnostic data from ELISA, IFAT, HmT, PCR, and qPCR techniques for chronic CD (Figure 9), due to differences in sensitivity and specificity, which can be generated by implicit or explicit variations across studies and variation in test cut-off points. The area under the curve (AUC) calculated for ELISA, IFAT, HmT, PCR, and qPCR was 0.989, 0.770, 0.988, 0.957, and 0.981, respectively, indicating slightly better performance for the ELISA in chronic CD. Likewise, when the AUC was restricted to the observed false positive rates (FPR) (AUC_FPR_) the results showed a relatively better performance of the ELISA diagnostic test for chronic CD (Figure 9). Additionally, data from ELISA and qPCR for acute CD were compared by sROC curve analysis. The AUC calculated for ELISA and qPCR was 0.986 and 0.987, respectively, indicating the similar performance of both techniques. However, when the AUC_FPR_ was calculated, the results showed a better performance of the ELISA diagnostic test for acute CD (Figure 10).

## 4. Discussion

CD was once confined to rural areas in Latin America, where it was commonly transmitted through vectors [108], while in recent years; increased migration has been pointed out as the main driver of the urbanization of the disease, which is a broad and complex response to changing labor needs and agricultural, demographic, and geopolitical conditions [109]. Therefore, the migratory flow has been key to the appearance of CD in areas where it was not previously reported, which makes CD a global concern that expands its geographical location in an increasingly globalized world [110]. In addition, it is estimated that 90% of people with CD are unaware of their infection and are therefore at risk of transmitting the disease and suffering from complications [111], which is aggravated since the dynamics of parasitemia during infection fluctuate [112]. Furthermore, diagnostic techniques employed in diagnosing CD have insufficient accessibility, sensitivity, specificity, and applicability, and most of them require expensive resources and equipment that are often unavailable in endemic areas [25,29,87]. Additionally, it has been proposed that the sensitivity and specificity of current tests are lower than generally reported in quality and unblinded studies, leading to the potential for underdiagnosis [113]. For these reasons, The Pan American Health Organization (PAHO) and the WHO developed a Guide for the Diagnosis and Treatment of Chagas Disease [114], which provides strategies for the timely diagnosis of CD, whereas the use of direct parasitological tests and eventual serological follow-up; and IgG-based serological assays (at least two immunological tests with different technical principles when a single assay does not reach the required accuracy) are currently recommended for acute and chronic CD diagnosis, respectively [115]. 

In recent times, molecular methods, such as PCR and qPCR, have been introduced as supportive diagnostic tests for CD [116], these methods represent a great advance in DNA and/or RNA quantification in biological samples and have been extensively studied in the assessment of the parasitic load in [117,118]; and have applied to adult and pediatric patients with suspected CD, exposure to *T. cruzi*, or a confirmed diagnosis in conditions such as the early diagnosis of congenital transmission in newborns, the diagnosis of oral infections, the early detection of infection in receptors of organs from CD donors, the monitoring of reactivation in chronically infected patients immune-suppressed due to transplantation or acquired immunodeficiency syndrome (AIDS) and the evaluation of treatment response [116]. Apart from that, the analysis presented in the current work of qPCR data shows the best performance among molecular diagnostic methods, with a median sensitivity and specificity of 94.0 and 98.0%, respectively. It has been stated that the diagnostic efficacy of molecular techniques is high in the acute CD, while in the chronic phase the immunological techniques are more effective [119]; when comparing the ELISA to qPCR methods in the diagnosis of the acute CD results, it was shown no significant difference; but, when comparing the AUC_FPR_, qPCR showed inferior results; which can be explained partially by the difference in the number of studies evaluated and sample sizes. Additionally, the target product profiles (TPPs) for molecular diagnosis of CD have been proposed for acute CD [20]. *T. cruzi* qPCR has been analytically validated, but it has not yet been clinically validated to assess its clinical utility [120], while recently the combined use of serological techniques and qPCR allowed identifying the highest prevalence of CD in humans, compared to the use of only one of these screening tools [121].

ELISA, HmT, and IFAT are standard techniques applied to detect anti-*T. cruzi* antibodies, whereas these diagnostic methods have different antibody recognition rationales [113]; however, ELISA exhibits several advantages over other techniques because of its simplicity, selectivity, and sensitivity [122]; besides, the PAHO recommendations and other diagnostic guidelines advise the use of two different serological techniques for chronic CD diagnosis, one of the techniques being ELISA [123]; for these reasons, several ELISA-based diagnostic tests have been developed and approved by the United States Food and Drug Administration (FDA) for blood donor screening and clinical diagnostic testing, [124]. Regarding the results obtained in the present work, the ELISA method presented the best performance among immunological methods with a median sensitivity and specificity of 99.0 and 99.0%, respectively. Furthermore, compared to other diagnosis methods accessed in the work, ELISA showed the best results in diagnosing acute and chronic CD, when the AUC was restricted to the observed false positive rates.

Although Xenodiagnosis allows CD to be diagnosed at the subclinical stage of the disease, where there are no clinical signs [28]; and those serological tests, such as CFT, RIPA, and WB are preferably used for the diagnosis of CD during the chronic phase [125]; unexpectedly, the number of studies selected made it impossible to include them in the meta-analysis, which requires at least 5 studies for analysis with a *p* < 0.05 [35]. Yet, a search of single MeSH terms for *“Chagas Disease”*, *“Sensitivity and Specificity”*, “*Xenodiagnosis”*, *“Complement Fixation Test”*, *“Radioimmunoprecipitation assay”* and *“Blotting, Western”* showed 14,253, 633,656, 117, 16,780, 566, 163,653 studies, respectively, while combining them only 15, 7, 8 and 15 were found, correspondingly. Inherent flaws associated with a systematic review and meta-analysis studies, such: as the location and selection of studies, loss of information on important outcomes, inappropriate subgroup analyses, conflict with new experimental data, and duplication of publication [126], should be considered as limitations of the present work. Additionally, across the studies analyzed, one of the main problems found was the heterogeneity of groups studied, clinical settings, and diagnostic performance measurements, whereas biased estimates of sensitivity and specificity, which could tend to inflate estimates, were also common problems found in the analyzed studies.

## 5. Conclusions

The accurate and sensitive diagnosis of CD is important for effective treatment and the adoption of control measures against the disease. This study found that the ELISA technique showed better diagnostic performance in the chronic and acute phases of CD compared to other serological (HmT and IFAT) and molecular (PCR and qPCR) techniques, suggesting its feasibility to be used for sensitive and specific CD diagnosis.

## Data Availability

Not applicable.

## Supplementary Materials

The following supporting information can be downloaded at: https://www.mdpi.com/article/10.3390/diagnostics12112752/s1, Figure S1: Study data and paired forest plot of the Positive Likelihood ratio, Negative likelihood ratio, and Diagnostic Odds ratio of Enzyme-linked immunosorbent assay (ELISA) in Chagas’s disease diagnosis; Figure S2: Study data and paired forest plot of the Positive Likelihood ratio, Negative likelihood ratio, and Diagnostic Odds ratio of Fluorescence antibody assay (IFAT) in Chagas’s disease diagnosis; Figure S3: Study data and paired forest plot of the Positive Likelihood ratio, Negative likelihood ratio, and Diagnostic Odds ratio Hemagglutination test (HmT) in Chagas’s disease diagnosis; Figure S4: Study data and paired forest plot of the Positive Likelihood ratio, Negative likelihood ratio, and Diagnostic Odds ratio Polymerase chain reaction in Chagas’s disease diagnosis; Figure S5: Study data and paired forest plot of the Positive Likelihood ratio, Negative likelihood ratio, and Diagnostic Odds ratio quantitative Polymerase chain reaction in Chagas’s disease diagnosis. Table S1: PRISMA 2020 Checklist.

## Abbreviations

The following abbreviations are used in this manuscript.

AIDS	Acquired Immunodeficiency Syndrome
AUC	Area Under the Curve
AUC_FPR_	Area Under the Curve Restricted to The False Positive Rates
BNZ	Benznidazole
CD	Chagas Disease
CFT	Complement Fixation Test
DOR	Diagnostic Likelihood Ratio
ELISA	Enzyme-Linked Immunosorbent Assay
FDA	Food and Drug Administration
HmT	Hemagglutination Test
IFAT	Fluorescent Antibody Technique
INPLASY	International Platform of Registered Systematic Review and Meta-analysis Protocols
LR−	Negative Likelihood Ratio
LR+	Positive Likelihood Ratio
MeSH	Medical Subject Headings
NCBI	National Center for Biotechnology Information
NFX	Nifurtimox
PAHO	Pan American Health Organization
PCR	Polymerase Chain Reaction
PRISMA	Preferred Reporting Items for Systematic Reviews and Meta-Analyses
qPCR	Real-Time Polymerase Chain Reaction
RIPA	Radioimmunoprecipitation Assay
ROC	Receiver Operating Characteristics
sROC	Summary Receiver Operating Characteristics
TPPs	Target Product Profiles
WB	Western Blot
WHO	World Health Organization

## References

[1] Kratz J.M. (2019). Drug discovery for Chagas disease: A viewpoint. Acta Trop..

[2] Ribeiro V., Dias N., Paiva T., Hagström-Bex L., Nitz N., Pratesi R., Hecht M. (2020). Current trends in the pharmacological management of Chagas disease. Int. J. Parasitol. Drugs Drug Resist..

[3] Punukollu G., Gowda R.M., Khan I.A., Navarro V.S., Vasavada B.C. (2007). Clinical aspects of the Chagas’ heart disease. Int. J. Cardiol..

[4] Pérez-Molina J.A., Molina I. (2018). Chagas disease. Lancet.

[5] Martínez-Peinado N., Cortes-Serra N., Losada-Galvan I., Alonso-Vega C., Urbina J.A., Rodríguez A., VandeBerg J.L., Pinazo M.-J., Gascon J., Alonso-Padilla J. (2020). Emerging agents for the treatment of Chagas disease: What is in the preclinical and clinical development pipeline?. Expert Opin. Investig. Drugs.

[6] Pinazo M.J., Pereiro A., Herazo R., Chopita M., Forsyth C., Lenardón M., Losada I., Torrico F., Marchiol A., Vera M. (2020). Interventions to bring comprehensive care to people with Chagas disease: Experiences in Bolivia, Argentina and Colombia. Acta Trop..

[7] Irish A., Whitman J.D., Clark E.H., Marcus R., Bern C. (2022). Updated estimates and mapping for prevalence of Chagas disease among adults, United States. Emerg. Infect. Dis..

[8] Hotez P.J., Dumonteil E., Betancourt Cravioto M., Bottazzi M.E., Tapia-Conyer R., Meymandi S., Karunakara U., Ribeiro I., Cohen R.M., Pecoul B. (2013). An unfolding tragedy of Chagas disease in North America. PLoS Negl. Trop. Dis..

[9] Villalta F., Rachakonda G. (2019). Advances in preclinical approaches to Chagas disease drug discovery. Expert Opin. Drug Discov..

[10] Casulli A. (2021). New global targets for NTDs in the WHO roadmap 2021–2030. PLoS Negl. Trop. Dis..

[11] Vermelho A.B., Rodrigues G.C., Supuran C.T. (2020). Why hasn’t there been more progress in new Chagas disease drug discovery?. Expert Opin. Drug Discov..

[12] Ribeiro A.L., Nunes M.P., Teixeira M.M., Rocha M.O.C. (2012). Diagnosis and management of Chagas disease and cardiomyopathy. Nat. Rev. Cardiol..

[13] Suárez C., Nolder D., García-Mingo A., Moore D.A., Chiodini P.L. (2022). Diagnosis and clinical management of Chagas disease: An increasing challenge in non-endemic areas. Res. Rep. Trop. Med..

[14] Lidani K.C.F., Andrade F.A., Bavia L., Damasceno F.S., Beltrame M.H., Messias-Reason I.J., Sandri T.L. (2019). Chagas disease: From discovery to a worldwide health problem. Front. Public Health.

[15] Rangel-Gamboa L., López-García L., Moreno-Sánchez F., Hoyo-Ulloa I., Vega-Mémije M.E., Mendoza-Bazán N., Romero-Valdovinos M., Olivo-Díaz A., Villalobos G., Martínez-Hernández F. (2019). Trypanosoma cruzi infection associated with atypical clinical manifestation during the acute phase of the Chagas disease. Parasites Vectors.

[16] Echavarría N.G., Echeverría L.E., Stewart M., Gallego C., Saldarriaga C. (2021). Chagas disease: Chronic Chagas cardiomyopathy. Curr. Probl. Cardiol..

[17] Córdova E., Maiolo E., Corti M., Orduña T. (2010). Neurological manifestations of Chagas’ disease. Neurol. Res..

[18] Sosa-Estani S., Segura E.L. (2015). Integrated control of Chagas disease for its elimination as public health problem—A review. Mem. Inst. Oswaldo Cruz.

[19] Pinheiro E., Brum-Soares L., Reis R., Cubides J.-C. (2017). Chagas disease: Review of needs, neglect, and obstacles to treatment access in Latin America. Rev. Soc. Bras. Med. Trop..

[20] Porrás A.I., Yadon Z.E., Altcheh J., Britto C., Chaves G.C., Flevaud L., Martins-Filho O.A., Ribeiro I., Schijman A.G., Shikanai-Yasuda M.A. (2015). Target product profile (TPP) for Chagas disease point-of-care diagnosis and assessment of response to treatment. PLoS Negl. Trop. Dis..

[21] Martinez S.J., Romano P.S., Engman D.M. (2020). Precision health for Chagas disease: Integrating parasite and host factors to predict outcome of infection and response to therapy. Front. Cell. Infect. Microbiol..

[22] Alcolea V., Pérez-Silanes S. (2020). Selenium as an interesting option for the treatment of Chagas disease: A review. Eur. J. Med. Chem..

[23] Dumonteil E., Herrera C., Buekens P. (2019). A therapeutic preconceptional vaccine against Chagas disease: A novel indication that could reduce congenital transmission and accelerate vaccine development. PLoS Negl. Trop. Dis..

[24] Michel-Todó L., Reche P.A., Bigey P., Pinazo M.-J., Gascón J., Alonso-Padilla J. (2019). In silico design of an epitope-based vaccine ensemble for Chagas disease. Front. Immunol..

[25] Lozano D., Rojas L., Méndez S., Casellas A., Sanz S., Ortiz L., Pinazo M.J., Abril M., Gascón J., Torrico F. (2019). Use of rapid diagnostic tests (RDTs) for conclusive diagnosis of chronic Chagas disease—Field implementation in the Bolivian Chaco region. PLoS Negl. Trop. Dis..

[26] Moure Z., Angheben A., Molina I., Gobbi F., Espasa M., Anselmi M., Salvador F., Tais S., Sánchez-Montalvá A., Pumarola T. (2016). Serodiscordance in chronic Chagas disease diagnosis: A real problem in non-endemic countries. Clin. Microbiol. Infect..

[27] Piron M., Fisa R., Casamitjana N., López-Chejade P., Puig L., Vergés M., Gascón J., Gomez i Prat J., Portús M., Sauleda S. (2007). Development of a real-time PCR Assay for Trypanosoma Cruzi Detection in Blood Samples. Acta Trop..

[28] Sánchez-Vega J.T., Almanza-Mackintoy A., Luna-Santillán A.V., de La Sancha-Solares T. (2020). A case report of Chagas disease in acute phase diagnosed by xenodiagnosis. Parasitol. Int..

[29] Alonso-Padilla J., Cortés-Serra N., Pinazo M.J., Bottazzi M.E., Abril M., Barreira F., Sosa-Estani S., Hotez P.J., Gascón J. (2019). Strategies to enhance access to diagnosis and treatment for Chagas disease patients in Latin America. Expert Rev. Anti-Infect. Ther..

[30] Brossas J.-Y., Griselda B., Bisio M., Guihenneuc J., Gulin J.E.N., Jauréguiberry S., Lescure F.-X., Fekkar A., Mazier D., Altcheh J. (2021). Evaluation of the Chagas western blot IgG assay for the diagnosis of Chagas disease. Pathogens.

[31] Picado A., Angheben A., Marchiol A., Alarcón de Noya B., Flevaud L., Pinazo M.J., Gállego M., Meymandi S., Moriana S. (2017). Development of diagnostics for Chagas disease: Where should we put our limited resources?. PLoS Negl. Trop. Dis..

[32] Balouz V., Agüero F., Buscaglia C.A. (2017). Chagas disease diagnostic applications: Present knowledge and future steps. Adv. Parasitol..

[33] Buttenheim A.M., Levy M.Z., Castillo-Neyra R., McGuire M., Toledo Vizcarra A.M., Mollesaca Riveros L.M., Meza J., Borrini-Mayori K., Naquira C., Behrman J. (2019). A behavioral design approach to improving a Chagas disease vector control campaign in Peru. BMC Public Health.

[34] Page M.J., Moher D., Bossuyt P.M., Boutron I., Hoffmann T.C., Mulrow C.D., Shamseer L., Tetzlaff J.M., Akl E.A., Brennan S.E. (2021). PRISMA 2020 explanation and elaboration: Updated guidance and exemplars for reporting systematic reviews. Br. Med. J..

[35] Chávez-Fumagalli M.A., Shrivastava P., Aguilar-Pineda J.A., Nieto-Montesinos R., Del-Carpio G.D., Peralta-Mestas A., Caracela-Zeballos C., Valdez-Lazo G., Fernandez-Macedo V., Pino-Figueroa A. (2021). Diagnosis of Alzheimer’s disease in developed and developing countries: Systematic review and meta-analysis of diagnostic test accuracy. J. Alzheimers Dis. Rep..

[36] Hotez P., Bottazzi M.E., Strub-Wourgaft N., Sosa-Estani S., Torrico F., Pajín L., Abril M., Sancho J. (2020). A new patient registry for Chagas disease. PLoS Negl. Trop. Dis..

[37] Kim S., Yeganova L., Wilbur W.J. (2016). Meshable: Searching PubMed abstracts by utilizing MeSH and MeSH-derived topical terms. Bioinformatics.

[38] White J. (2020). PubMed 2.0. Med. Ref. Serv. Q..

[39] Van Eck N.J., Waltman L. (2017). Citation-based clustering of publications using CitNetExplorer and VOSviewer. Scientometrics.

[40] Waltman L., Van Eck N.J. (2012). A new methodology for constructing a publication-level classification system of science. J. Assoc. Inf. Sci. Technol..

[41] Lee J., Kim K.W., Choi S.H., Huh J., Park S.H. (2015). Systematic review and meta-analysis of studies evaluating diagnostic test accuracy: A practical review for clinical researchers–part II. Statistical methods of meta-analysis. Korean J. Radiol..

[42] Shim S.R., Kim S.-J., Lee J. (2019). Diagnostic test accuracy: Application and practice using R software. Epidemiol. Health.

[43] Shreffler J., Huecker M.R. (2022). Diagnostic testing accuracy: Sensitivity, specificity, predictive values and likelihood ratios. StatPearls [Internet].

[44] Huang Y., Yin J., Samawi H. (2018). Methods improving the estimate of diagnostic odds ratio. Commun. Stat. Simul. Comput..

[45] Reitsma J.B., Glas A.S., Rutjes A.W.S., Scholten R.J.P.M., Bossuyt P.M., Zwinderman A.H. (2005). Bivariate analysis of sensitivity and specificity produces informative summary measures in diagnostic reviews. J. Clin. Epidemiol..

[46] Requejo H.I., Nakamura P.M., Vaz A.J., Pialarissi C.S., Hoshino-Shimizu S., Matsumoto T.K., Nakamura H. (1991). Diffusion-in-gel enzyme-linked immunosorbent assay (DIG-ELISA) for Chagas’ disease serodiagnosis. Braz. J. Med. Biol. Res..

[47] Solana M.E., Katzin A.M., Umezawa E.S., Miatello C.S. (1995). High specificity of Trypanosoma cruzi epimastigote ribonucleoprotein as antigen in serodiagnosis of Chagas’ disease. J. Clin. Microbiol..

[48] Peralta J.M., Teixeira M.D.G.M., Shreffler W.G., Pereira J.B., Burns J.M., Sleath P.R., Reed S.G. (1994). Serodiagnosis of Chagas’ disease by enzyme-linked immunosorbent assay using two synthetic peptides as antigens. J. Clin. Microbiol..

[49] Almeida I.C., Covas D.T., Soussumi L.M., Travassos L.R. (1997). A highly sensitive and specific chemiluminescent enzyme-linked immunosorbent assay for diagnosis of active Trypanosoma cruzi infection. Transfusion.

[50] Monteón V.M., Guzman-Rojas L., Negrete-Garcia C., Rosales-Encina J.L., Reyes-Lopez P.A. (1997). Serodiagnosis of american trypanosomosis by using nonpathogenic trypanosomatid antigen. J. Clin. Microbiol..

[51] Partel C.D., Rossi C.L. (1998). A rapid, quantitative enzyme-linked immunosorbent assay (ELISA) for the immunodiagnosis of Chagas’ disease. Immunol. Investig..

[52] Oelemann W.M.R., Teixeira M.D.G.M., Veríssimo Da Costa G.C., Borges-Pereira J., De Castro J.A.F., Coura J.R., Peralta J.M. (1998). Evaluation of three commercial enzyme-linked immunosorbent assays for diagnosis of Chagas’ disease. J. Clin. Microbiol..

[53] Pinho R.T., Pedrosa R.C., Costa-martins P., Castello-Branco L.R.R. (1999). Saliva ELISA: A method for the diagnosis of chronic Chagas disease in endemic areas. Acta Trop..

[54] Houghton R.L., Benson D.R., Reynolds L.D., McNeill P.D., Sleath P.R., Lodes M.J., Skeiky Y.A., Leiby D.A., Badaro R., Reed S.G. (1999). A multi-epitope synthetic peptide and recombinant protein for the detection of antibodies to Trypanosoma cruzi in radioimmunoprecipitation-confirmed and consensus-positive sera. J. Infect. Dis..

[55] Umezawa E.S., Bastos S.F., Camargo M.E., Yamauchi L.M., Santos M.R., Gonzalez A., Zingales B., Levin M.J., Sousa O., Rangel-Aldao R. (1999). Evaluation of recombinant antigens for serodiagnosis of Chagas’ disease in South and Central America. J. Clin. Microbiol..

[56] Betonico G.N., Miranda E.O., Silva D.A.O., Houghton R., Reed S.G., Campos-Neto A., Mineo J.R. (1999). Evaluation of a synthetic tripeptide as antigen for detection of IgM and IgG antibodies to Trypanosoma cruzi in serum samples from patients with Chagas disease or viral diseases. Trans. R. Soc. Trop. Med. Hyg..

[57] Pereira V.R.A., Nakazawa M., Furtado V.C., Abath F.G.C., Gomes Y.M. (2000). Immunodiagnosis of chronic Chagas’ disease using the Tc 46 and Tc 58 antigens. Rev. Soc. Bras. Med. Trop..

[58] Gomes Y.M., Pereira V.R., Nakazawa M., Rosa D.S., Barros M.d.N.D., Ferreira A.G., Silva E.D., Yamada S.F., Krieger M.A., Goldenberg S. (2001). Serodiagnosis of chronic Chagas infection by using EIE-recombinant-Chagas-biomanguinhos kit. Mem. Inst. Oswaldo Cruz.

[59] Blejer J.L., Saguier M.C., Salamone H.J. (2001). Antibodies to Trypanosoma cruzi among blood donors in Buenos Aires, Argentina. Int. J. Infect. Dis..

[60] Nakazawa M., Rosa D.S., Pereira V.R., Moura M.O., Furtado V.C., Souza W.V., Barros M., Abath F., Gomes Y.M. (2001). Excretory-secretory antigens of Trypanosoma cruzi are potentially useful for serodiagnosis of chronic Chagas’ disease. Clin. Diagn. Lab. Immunol..

[61] Sánchez B., Monteón V., Reyes P.A., Espinoza B. (2001). Standardization of micro-enzyme-linked immunosorbent assay (ELISA) and western blot for detection of Trypanosoma cruzi antibodies using extracts from Mexican strains as antigens. Arch. Med. Res..

[62] Umezawa E.S., Bastos S.F., Coura J.R., Levin M.J., Gonzalez A., Rangel-Aldao R., Zingales B., Luquetti A., da Silveira J.F. (2003). An improved serodiagnostic test for Chagas’ disease employing a mixture of Trypanosoma cruzi recombinant antigens. Transfusion.

[63] Gadelha A.Á.M., Verçosa A.F.A., Lorena V.M.B., Nakazawa M., Carvalho A.B., Souza W.V., Ferreira A.G.P., Silva E.D., Krieger M.A., Goldenberg S. (2003). Chagas’ disease diagnosis: Comparative analysis of recombinant ELISA with conventional ELISA and the haemagglutination test. Vox. Sang..

[64] Umezawa E.S., Luquetti A.O., Levitus G., Ponce C., Ponce E., Henriquez D., Revollo S., Espinoza B., Sousa O., Khan B. (2004). Serodiagnosis of chronic and acute Chagas’ disease with Trypanosoma cruzi recombinant protein: Results of a collaborative study in six Latin American countries. J. Clin. Microbiol..

[65] Saldaña A., Samudio F., Miranda A., Herrera L.M., Saavedra S.P., Cáceres L., Bayard V., Calzada J.E. (2005). Predominance of Trypanosoma rangeli infection in children from a Chagas disease endemic area in the west-shore of the Panama canal. Mem. Inst. Oswaldo Cruz.

[66] Vieira Duarte A.M., Monteiro de Andrade H., Hadad do Monte S.J., Coelho Peixoto de Toledo V.d.P., Pinto Dabés Guimaraes T.M. (2006). Assessment of chemiluminescence and PCR effectiveness in relation to conventional serological tests for the diagnosis of Chagas’ disease. Rev. Soc. Bras. Med. Trop..

[67] Ramírez J.D., Guhl F., Umezawa E.S., Morillo C.A., Rosas F., Marin-Neto J.A., Restrepo S. (2009). Evaluation of adult chronic Chagas’ heart disease diagnosis by molecular and serological methods. J. Clin. Microbiol..

[68] Mateo H., Sánchez-Moreno M., Marín C. (2010). Enzyme-linked immunosorbent assay with purified Trypanosoma cruzi excreted superoxide dismutase. Clin. Biochem..

[69] Briceño L., Rodríguez E.M., Medina M., Campos Y., Mosca W., Briceño A., León G. (2010). An inexpensive antigen for serodiagnosis of Chagas’ disease. Investig. Clin..

[70] de Marchi C.R., Di Noia J.M., Frasch A.C.C., Neto V.A., Almeida I.C., Buscaglia C.A. (2011). Evaluation of a recombinant Trypanosoma cruzi mucin-like antigen for serodiagnosis of Chagas’ disease. Clin. Vaccine Immunol..

[71] da Silva Santos L., Morais Torres R., Machado-de-Assis G.F., Bahia M.T., Rodrigues Martins H., Teixeira-Carvalho A., Alves Coelho-dos-Reis J.G., Albajar-Viñas P., Olindo M.-F., de Lana M. (2012). In-house ELISA method to analyze anti-trypanosoma cruzi IgG reactivity for differential diagnosis and evaluation of Chagas disease morbidity. Rev. Soc. Bras. Med. Trop..

[72] López-Céspedes Á., Villagrán E., Briceño Álvarez K., de Diego J.A., Hernández-Montiel H.L., Saldaña C., Sánchez-Moreno M., Marín C. (2012). Trypanosoma cruzi: Seroprevalence detection in suburban population of Santiago de Querétaro (Mexico). Sci. World J..

[73] Longhi S.A., Brandariz S.B., Lafon S.O., Niborski L.L., Luquetti A.O., Schijman A.G., Levin M.J., Gómez K.A. (2012). Short report: Evaluation of in-house ELISA using Trypanosoma cruzi lysate and recombinant antigens for diagnosis of chagas disease and discrimination of its clinical forms. Am. J. Trop. Med. Hyg..

[74] Tustin A.W., Small D.S., Delgado S., Castillo Neyra R., Verastegui M.R., Ancca Juárez J.M., Quispe Machaca V.R., Gilman R.H., Bern C., Levy M.Z. (2012). Use of individual-level covariates to improve latent class analysis of Trypanosoma cruzi diagnostic tests. Epidemiol. Methods.

[75] Marín C., Concha-Valdez F., Cañas R., Gutiérrez-Sánchez R., Sánchez-Moreno M. (2014). Anti-Trypanosoma cruzi antibody detection in Eastern Andalusia (Spain). Trans. R. Soc. Trop. Med. Hyg..

[76] Duarte L.F., Flórez O., Rincón G., González C.I. (2014). Comparison of seven diagnostic tests to detect Trypanosoma cruzi infection in patients in chronic phase of Chagas disease. Colomb. Med..

[77] Dopico E., Pimenta Del-rei R., Espinoza B., Ubillos I., Tonin Zanchin N.I., Sulleiro E., Moure Z., Fiorani Celedon P.A., Vieira Souza W., Domingos da Silva E. (2019). Immune reactivity to Trypanosoma cruzi chimeric proteins for Chagas disease diagnosis in immigrants living in a non-endemic setting. BMC Infect. Dis..

[78] Caballero E.Z., Correa R., Nascimento M.S., Villarreal A., Llanes A., Kesper J.R.N. (2019). High sensitivity and reproducibility of in-house ELISAs using different genotypes of Trypanosoma cruzi. Parasite Immunol..

[79] Floridia-Yapur N., Monje-Rumi M., Ragone P., Lauthier J.J., Tomasini N., D’Amato A.A., Diosque P., Cimino R., Gil J., Sanchez D. (2010). TcTASV antigens of Trypanosoma cruzi: Utility for diagnosis and high accuracy as biomarkers of treatment efficacy in pediatric patients. Am. J. Trop. Med. Hyg.

[80] Abramo C., Fontes C.J., Krettli A.U. (1995). Cross-reactivity between antibodies in the sera of individuals with leishmaniasis, toxoplasmosis, and Chagas’ disease and Antigens of the blood-stage forms of plasmodium falciparum determined by indirect immunofluorescence. Am. J. Trop. Med. Hyg..

[81] Matsumoto T.K., Hoshino-Shimizu S., Nakamura P.M., Andrade H.F., Umezawa E.S. (1993). High resolution of Trypanosoma cruzi amastigote antigen in serodiagnosis of different clinical forms of Chagas’ disease. J. Clin. Microbiol..

[82] de Apparecida Levy A.M., Boainain E., Kloetzel J.K. (1996). In situ indirect fluorescent antibody: A new specific test to detect ongoing chagasic infections. J. Clin. Lab. Anal..

[83] Langhi D.M., Bordin J.O., Castelo A., Walter S.D., Moraes-Souza H., Stumpf R.J. (2002). The application of latent class analysis for diagnostic test validation of chronic Trypanosoma cruzi infection in blood donors. Braz. J. Infect. Dis..

[84] Bergmann Araújo A., Aires Berne M.E. (2013). Conventional Serological Performance in Diagnosis of Chagas’ Disease in Southern Brazil. Braz. J. Infect. Dis..

[85] Blejer J.L., Saguier M.C., Dinapoli R.A., Salamone H.J. (1999). Prevalence of Trypanosoma cruzi antibodies in blood donors. Medicina.

[86] Remesar M.C., Gamba C., Colaianni I.F., Puppo M., Sartor P.A., Murphy E.L., Neilands T.B., Ridolfi M.A., Leguizamón M.S., Kuperman S. (2010). Estimation of sensitivity and specificity of several Trypanosoma cruzi antibody assays in blood donors in Argentina. Transfusion.

[87] Egüez K.E., Alonso-Padilla J., Terán C., Chipana Z., García W., Torrico F., Gascon J., Lozano-Beltran D.-F., Pinazo M.-J. (2017). Rapid diagnostic tests duo as alternative to conventional serological assays for conclusive Chagas disease diagnosis. PLoS Negl. Trop. Dis..

[88] Barbosa Marcon G.E., Durante Andrade P., de Albuquerque D.M., da Silva Wanderley J., de Almeida E.A., Guariento M.E., Botelho Costa S.C. (2002). (2002). Use of a nested polymerase chain reaction (N-PCR) to detect Trypanosoma cruzi in blood samples from chronic chagasic patients and patients with doubtful serologies. Diagn. Microbiol. Infect. Dis..

[89] Altcheh J., Biancardi M., Lapena A., Ballering G., Freilij H. (2005). Congenital Chagas disease: Experience in the hospital de niños, Ricardo Gutierrez, Buenos Aires, Argentina. Rev. Soc. Bras. Med. Trop..

[90] Fitzwater S., Calderon M., LaFuente C., Galdos-Cardenas G., Ferrufino L., Verastegui M., Gilman R.H., Bern C. (2008). Short report: Polymerase chain reaction for chronic Trypanosoma cruzi infection yields higher sensitivity in blood clot than buffy coat or whole blood specimens. Am. J. Trop. Med. Hyg..

[91] Deborggraeve S., Coronado X., Solari A., Zulantay I., Apt W., Mertens P., Laurent T., Leclipteux T., Stessens T., Dujardin J.-C. (2009). T. Cruzi OligoC-TesT: A simplified and standardized polymerase chain reaction format for diagnosis of Chagas disease. PLoS Negl. Trop. Dis..

[92] Sabino E.C., Lee T.H., Montalvo L., Nguyen M.L., Leiby D.A., Carrick D.M., Otani M.M., Vinelli E., Wright D., Stramer S.L. (2013). Antibody levels correlate with detection of Trypanosoma cruzi DNA by sensitive polymerase chain reaction assays in seropositive blood donors and possible resolution of infection over time. Transfusion.

[93] Gilber S.R., Alban S.M., Gobor L., De Oliveira Bescrovaine J., Myiazaki M.I., Thomaz-Soccol V. (2013). Comparison of conventional serology and PCR methods for the routine diagnosis of Trypanosoma cruzi infection. Rev. Soc. Bras. Med. Trop..

[94] Hernández C., Cucunubá Z., Flórez C., Olivera M., Valencia C., Zambrano P., León C., Ramírez J.D. (2016). Molecular diagnosis of Chagas disease in Colombia: Parasitic loads and discrete typing units in patients from acute and chronic phases. PLoS Negl. Trop. Dis..

[95] Hernández C., Teherán A., Flórez C., Ramírez J.D. (2018). Comparison of parasite loads in serum and blood samples from patients in acute and chronic phases of Chagas disease. Parasitology.

[96] Seiringer P., Pritsch M., Flores-Chavez M., Marchisio E., Helfrich K., Mengele C., Hohnerlein S., Bretzel G., Löscher T., Hoelscher M. (2017). Comparison of four PCR methods for efficient detection of Trypanosoma cruzi in routine diagnostics. Diagn. Microbiol. Infect. Dis..

[97] Cura C.I., Ramírez J.C., Rodríguez M., Lopez-Albízu C., Irazu L., Scollo K., Sosa-Estani S. (2017). Comparative study and analytical verification of PCR methods for the diagnosis of congenital Chagas disease. J. Mol. Diagn..

[98] Melo M.F., Moreira O.C., Tenório P., Lorena V., Lorena-Rezende I., Oliveira Júnior W., Gomes Y., Britto C. (2015). Usefulness of real time PCR to quantify parasite load in serum samples from chronic Chagas disease patients. Parasites Vectors.

[99] Mayta H., Romero Y.K., Pando A., Verastegui M., Tinajeros F., Bozo R., Henderson-Frost J., Colanzi R., Flores J., Lerner R. (2019). Improved DNA extraction technique from clot for the diagnosis of Chagas disease. PLoS Negl. Trop. Dis..

[100] Ramírez J.D., Herrera G., Hernández C., Cruz-Saavedra L., Muñoz M., Flórez C., Butcher R. (2018). Evaluation of the analytical and diagnostic performance of a digital droplet polymerase chain reaction (DdPCR) assay to detect Trypanosoma cruzi DNA in blood samples. PLoS Negl. Trop. Dis..

[101] Saavedra M., Zulantay I., Apt W., Martínez G., Rojas A., Rodríguez J. (2013). Chronic Chagas disease: PCR-xenodiagnosis without previous microscopic observation is a useful tool to detect viable Trypanosoma cruzi. Biol. Res..

[102] Garcia E., Ramirez L.E., Monteon V., Sotelo J. (1995). Diagnosis of American trypanosomiasis (Chagas’ disease) by the new complement fixation test. J. Clin. Microbiol..

[103] Winkler M.A., Brashear R.J., Hall H.J., Schur J.D., Pan A.A. (1995). Detection of antibodies to Trypanosoma cruzi among blood donors in the southwestern and western United States. II. Evaluation of a supplemental enzyme immunoassay and radioimmunoprecipitation assay for confirmation of seroreactivity. Transfusion.

[104] Verani J.R., Seitz A., Gilman R.H., Lafuente C., Galdos-cardenas G., Kawai V., Lafuente E.D., Ferrufino L., Bowman N.M., Pinedo-Cancino V. (2009). Geographic variation in the sensitivity of recombinant antigen-based rapid tests for chronic Trypanosoma cruzi infection. Am. J. Trop. Med. Hyg..

[105] Reiche E.M., Cavazzana Jr M., Okamura H., Tagata E.C., Jankevicius S.I., Jankevicius J.V. (1998). Evaluation of the western blot in the confirmatory serologic diagnosis of Chagas’ disease. Am. J. Trop. Med. Hyg..

[106] Caballero Z.C., Sousa O.E., Marques W.P., Saez-Alquezar A., Umezawa E.S. (2007). Evaluation of serological tests to identify Trypanosoma cruzi infection in humans and determine cross-reactivity with Trypanosoma rangeli and Leishmania spp. Clin. Vaccine Immunol..

[107] Otani M.M., Vinelli E., Kirchhoff L.V., del Pozo A., Sands A., Vercauteren G., Sabino E.C. (2009). WHO comparative evaluation of serologic assays for Chagas disease. Transfusion.

[108] Martins-Melo F.R., Castro M.C., Werneck G.L. (2021). Levels and trends in Chagas disease-related mortality in Brazil, 2000–2019. Acta Trop..

[109] Adger W.N., Crépin A.-S., Folke C., Ospina D., Chapin F.S., Segerson K., Seto K.C., Anderies J.M., Barrett S., Bennett E.M. (2020). Urbanization, migration, and adaptation to climate change. One Earth.

[110] Guhl F., Ramírez J.D. (2021). Poverty, migration, and Chagas disease. Curr. Trop. Med. Rep..

[111] Angheben A., Buonfrate D., Cruciani M., Jackson Y., Alonso-Padilla J., Gascon J., Gobbi F., Giorli G., Anselmi M., Bisoffi Z. (2019). Rapid immunochromatographic tests for the diagnosis of chronic Chagas disease in at-risk populations: A systematic review and meta-analysis. PLoS Negl. Trop. Dis..

[112] Olivera M.J., Porras Villamil J.F., Toquica Gahona C.C., Rodríguez Hernández J.M. (2018). Barriers to diagnosis access for Chagas disease in Colombia. J. Parasitol. Res..

[113] Afonso A.M., Ebell M.H., Tarleton R.L. (2012). A systematic review of high quality diagnostic tests for Chagas disease. PLoS Negl. Trop. Dis..

[114] Organización Panamericana de la Salud (2020). Síntesis de evidencia: Guía para el diagnóstico y el tratamiento de la enfermedad de Chagas. Rev. Panam. Salud Publica.

[115] Schijman A.G., Alonso-Padilla J., Longhi S.A., Picado A. (2022). Parasitological, serological and molecular diagnosis of acute and chronic Chagas disease: From field to laboratory. Mem. Inst. Oswaldo Cruz.

[116] Schijman A.G. (2018). Molecular diagnosis of Trypanosoma cruzi. Acta Trop..

[117] D’Ávila D.A., Galvão L.M.C., Sousa G.R., Britto C., Moreira O.C., Chiari E. (2018). Monitoring the parasite load in chronic Chagas disease patients: Comparison between blood culture and quantitative real time PCR. PLoS ONE.

[118] Parrado R., Ramirez J.C., de la Barra A., Alonso-Vega C., Juiz N., Ortiz L., Illanes D., Torrico F., Gascon J., Alves F. (2019). Usefulness of serial blood sampling and PCR replicates for treatment monitoring of patients with chronic Chagas disease. Antimicrob. Agents Chemother..

[119] Ferrer E., Lares M., Viettri M., Medina M. (2013). Comparación entre técnicas inmunológicas y moleculares para el diagnóstico de la enfermedad de Chagas. Enferm. Infecc. Microbiol. Clin..

[120] Duffy T., Cura C.I., Ramirez J.C., Abate T., Cayo N.M., Parrado R., Diaz Bello Z., Velazquez E., Muñoz-Calderon A., Juiz N.A. (2013). Analytical performance of a multiplex Real-Time PCR assay using TaqMan probes for quantification of Trypanosoma cruzi satellite DNA in blood samples. PLoS Negl. Trop. Dis..

[121] Chan-Pérez J.I., Torres-Acosta J.F.J., Ortega-Pacheco A., Hernández-Cortazar I.B., Cigarroa-Toledo N., Jiménez-Coello M. (2022). Combined Use of Real-Time PCR and serological techniques for improved surveillance of chronic and acute American Trypanosomiasis in dogs and their owners from an endemic rural area of neotropical Mexico. Curr. Res. Parasitol. Vector-Borne Dis..

[122] Sakamoto S., Putalun W., Vimolmangkang S., Phoolcharoen W., Shoyama Y., Tanaka H., Morimoto S. (2018). Enzyme-linked immunosorbent assay for the quantitative/qualitative analysis of plant secondary metabolites. J. Nat. Med..

[123] Brasil P.E., de Castro L., Hasslocher-Moreno A.M., Sangenis L.H., Braga J.U. (2010). ELISA versus PCR for diagnosis of chronic Chagas disease: Systematic review and meta-analysis. BMC Infect. Dis..

[124] Rivera H.N., McAuliffe I., Aderohunmu T., Wiegand R.E., Montgomery S.P., Bradbury R.S., Handali S. (2022). Evaluation of the performance of ortho T. cruzi ELISA test system for the detection of antibodies to Trypanosoma cruzi. J. Clin. Microbiol..

[125] Elisei R.M.T., Matos C.S., Carvalho A.M.R.S., Chaves A.T., Medeiros F.A.C., Barbosa R., Marcelino A.P., dos Santos Emidio K., Coelho E.A.F., Duarte M.C. (2018). Immunogenomic screening approach to identify new antigens for the serological diagnosis of chronic Chagas’ disease. Appl. Microbiol. Biotechnol..

[126] Gopalakrishnan P., Ganeshkumar S. (2013). Systematic reviews and meta-analysis: Understanding the best evidence in primary healthcare. J. Fam. Med. Prim. Care.

